# The Antiemetic Effect of Xiao-Ban-Xia-Tang Formula against Cisplatin-Induced Emesis is Mediated through Inhibition of NLRP3 Inflammasome Activation in a Rat Pica Model

**DOI:** 10.1155/2020/5497078

**Published:** 2020-03-29

**Authors:** Qi Meng, QianQian Cheng, Xiaodi Feng, Siqi Chen, Yaqi Li, Guanglong Zhang, Ke Nie

**Affiliations:** ^1^School of Chinese Materia Medica, Guangdong Pharmaceutical University, Guangzhou 510006, China; ^2^School of Chinese Medicine, Shandong University of Traditional Chinese Medicine, Jinan 250355, China

## Abstract

Xiao-Ban-Xia-Tang (XBXT), a traditional Chinese medicine formula, has been used for the treatment of emesis for nearly 2000 years, but its underlying mechanism is not yet fully clarified. The purpose of this study is to reveal the antiemetic mechanisms of XBXT by focusing on the NLRP3 inflammasome pathway in a chemotherapy-induced rat pica model. The pica model was generated by a single intraperitoneal injection of cisplatin in this study. Consumption of kaolin (a type of clay) and food and body weight were recorded every 24 hours. Cisplatin-induced increase in kaolin consumption (pica) was used to quantify chemotherapy-induced nausea and vomiting (CINV). Tissue from the ileum and antrum was stained with hematoxylin eosin (HE) to observe pathological changes. The levels of reactive oxygen species (ROS) and inflammatory cytokines, including IL-1*β* and IL-18 in serum, were detected by ELISA. In addition, changes in the NLRP3 inflammasome activation in the ileum and antrum were investigated using western blot and immunofluorescence microscopy. The results showed that oral administration of XBXT and ondansetron inhibited acute and delayed pica and significantly protected against the gastrointestinal pathological injury induced by cisplatin. The levels of ROS, IL-1*β*, and IL-18 in the serum of cisplatin-treated rats were also remarkably decreased by XBXT and ondansetron. Moreover, we found that XBXT can inhibit cisplatin-induced NLRP3 inflammasome activation. The present study indicates that the inhibition of the NLRP3 inflammasome activation might be one of the potential mechanisms for the therapeutic effects of XBXT against CINV.

## 1. Introduction

Chemotherapy-induced nausea and vomiting (CINV) is among the most debilitating and distressing events in cytotoxic chemotherapy [1]. The emetic response to chemotherapy is classified into acute phase (emesis occurring in the first 24 h after chemotherapy administration) and delayed phase (emesis occurring more than 24 h after chemotherapy administration) [2]. CINV can result in serious complications, such as weight loss, electrolyte imbalances, dehydration, and anorexia [3], which undermine the efficacy of chemotherapy and increase cost of care. Thus, there is an urgent need for effective control of CINV. A variety of neurotransmitters and their receptors are involved in the development of CINV, but the underlying mechanisms remain mostly unclear [2]. Currently, for the treatment of nausea and vomiting in patients receiving high-emetic chemotherapy (HEC), the European Society of Medical Oncology (ESMO) and the Multinational Association of Supportive Care in Cancer (MASCC) guidelines [4] recommend a combination of a 5-hydroxytryptamine type 3 receptor antagonist (5-HT_3_RA, such as ondansetron), dexamethasone, and aprepitant administered before chemotherapy to prevent acute nausea and vomiting and dexamethasone plus aprepitant or aprepitant alone can be used to prevent delayed nausea and vomiting. However, these agents do not completely resolve CINV. Despite the availability of effective antiemetic drugs, control of CINV is suboptimal [5]. At present, there is an unmet need for safer and less expensive antiemetic drugs.

Cisplatin, a platinum-based chemotherapeutic drug that has been used worldwide to treat various types of cancer, is classified as a HEC [4, 6]. Although cisplatin is highly effective against tumors, it is also associated with gastrointestinal toxicity [7], such as gastrointestinal inflammation [8]. Moreover, extreme nausea and vomiting are the most frequent adverse side effects of cisplatin [9]. However, a causal relationship between cisplatin-induced gastrointestinal inflammation and cisplatin-induced emesis has not been established. The etiology and pathogenesis of cisplatin-induced CINV are complex, but numerous studies have shown that inflammation plays a role in the pathological processes that underlie CINV [10, 11].

5-HT_3_RAs are the most commonly used antiemetics for preventing CINV in clinic. It was reported that ramosetron and ondansetron could improve the pathological changes of the small intestine and inhibit the increase in the levels of TNF-*α*, IL-1*β*, and IL-6 in a chemotherapy-treated mouse model. The above results suggested that 5-HT_3_RAs may be useful for preventing not only CINV but also the intestinal inflammatory response during chemotherapy [12]. Dexamethasone, a steroid anti-inflammatory drug, is commonly used to prevent and treat CINV recommended by ESMO and MASCC guidelines, which is related to its significant anti-inflammatory effect [13]. Thalidomide, which has potent anti-inflammatory activity [14], could attenuate cisplatin-induced pica in rats [15] and significantly improve HEC-induced delayed nausea and vomiting prevention in chemotherapy-naive patients [16]. These studies indicated that there is a close relationship between CINV and inflammation.

Smith et al. considered cancer treatment-related symptoms, a cluster of side effects that occur following the administration of chemotherapeutic agents, to be homologous to “sickness behavior,” which is initiated by the production and synergistic effect of TNF-*α* and IL-1*β* [17]. IL-1*β* is a key proinflammatory cytokine involved in chemotherapy-induced gastrointestinal toxicity [18], and IL-1*β* expression is increased in the rat's small intestine after cisplatin administration [8]. Reactive oxygen species (ROS) produced in response to cisplatin treatment not only initiates oxidative stress in the gut, but also triggers an inflammatory cascade involving nuclear factor kappa B (NF-*κ*B) signaling pathways that produce a variety of proinflammatory cytokines [8, 19]. The NOD-like receptor pyrin domain-containing protein 3 (NLRP3) inflammasome, one of the most well-characterized inflammasomes, is an oligomeric molecular complex that can be activated by various “danger signals” (e.g., ROS) and promote the maturation of IL-1*β* [20]. Upon detection of cellular stress, intracellular NLRP3 recruits apoptosis-associated speck-like protein (ASC), which contains a caspase recruitment domain and binds procaspase-1 to form the NLRP3 inflammasome. Assembly of the NLRP3 inflammasome promotes procaspase-1 self-cleavage to generate active caspase-1, which induces pro-IL-1*β* and pro-IL-18 maturation and secretion of IL-1*β* and IL-18 [21]. In fact, some studies have defined the NLRP3 inflammasome as a critical component in the pathogenesis and development of cisplatin-induced liver and kidney injury [22]. Therefore, we attempted to explore whether the NLRP3 inflammasome contributes to the pathogenesis of CINV triggered by cisplatin.

Xiao-Ban-Xia-Tang (XBXT) is a classic Chinese herbal formula for treating emesis and is composed of pinellia (*Pinellia ternata*) and ginger (*Zingiber officinale*). Use of XBXT was originally recorded in *Synopsis of Prescriptions of the Golden Chamber* written 2000 years ago. Animal experiments have revealed that XBXT inhibits cisplatin-induced acute and delayed emesis in minks, possibly by inhibiting central or peripheral increases in neurokinin-1 receptor levels [23]. XBXT also has good activity against cisplatin-induced kaolin consumption in rats, possibly by inhibiting central or peripheral increases in obestatin levels, or by inhibiting increases in the levels of cholecystokinin and calcitonin gene-related peptide in the blood [24]. Among the components of XBXT, ginger was found to be effective in reducing the severity of acute and delayed CINV as an additional therapy to ondansetron and dexamethasone in patients receiving HEC [25]. Gingerol, the generic term for pungent constituents in ginger, is effective against cisplatin-induced emesis in rats by inhibiting central or peripheral increases in dopamine (DA) levels [26]. 6-Gingerol is a natural compound extracted from ginger. 6-Gingerol significantly improved the overall complete response (CR) rate in CINV, appetite, and quality of life in cancer patients receiving adjuvant chemotherapy [27]. However, several components of XBXT identified by high-performance liquid chromatography (HPLC) have been demonstrated to have anti-inflammatory effects [28–30]. Among them, 6-shogaol has a potent capacity to attenuate canonical NLRP3 inflammasome-mediated IL-1*β* secretion in THP-1 macrophages [31]. However, the mechanism by which XBXT may affect inflammatory signal transduction during the progression of CINV remains largely unclarified.

In the present study, we investigated the potential effects of XBXT on CINV in a rat pica model. Specifically, we explored whether XBXT can protect against CINV by alleviating the inflammation states via suppressing NLRP3 inflammasome activation. We also use ondansetron as a comparator for the anti-inflammatory and antiemetic effects of XBXT against cisplatin.

## 2. Materials and Methods

### 2.1. Drugs and Reagents

Pinellia was produced in Xihe County, Gansu Province, and ginger was produced in Laiwu City, Shandong Province. 6-Gingerol and 6-shogaol were purchased from Chengdu Master Biotechnology Co., Ltd. (Chengdu, China). Ephedrine hydrochloride and succinic acid were purchased from the National Institutes for Food and Drug Control (Beijing, China). Cisplatin for injection and ondansetron hydrochloride injection were purchased from Qilu Pharmaceutical Co., Ltd. (Jinan, China). Kaolin and gum arabic power were purchased from Sinopharm Chemical Reagent Co., Ltd. (Shanghai, China).

### 2.2. Preparation of XBXT

XBXT consists of two herbs (Table 1). All herbal medicines were validated by Professor Jizhu Liu, according to the *Chinese Pharmacopeia* (Edition 2015). Voucher specimens (numbers are listed in Table 1) were retained at the Herbarium of School of Chinese Materia Medica, Guangdong Pharmaceutical University.

Pinellia and ginger were mixed at a ratio of 2 : 1 and immersed in distilled water (10 times their total weight) for 1 h. Subsequently, they were boiled for 1.5 h. Extraction was repeated by adding distilled water (8 times their weight) and boiling for another 1 h. Finally, the extracted solutions were mixed and concentrated by low-pressure evaporation below 60°C to a concentration of 0.16 g raw materials per milliliter.

### 2.3. High-Performance Liquid Chromatography (HPLC) Analysis of XBXT

The major chemical components of XBXT extract were identified by HPLC fingerprinting analysis. Ephedrine, succinic acid, 6-gingerol, and 6-shogaol were selected as standard substances. Chromatographic separation was performed on a WondaSil C18 (250 mm × 4.6 mm, 5 *μ*m). Chromatographic conditions were as follows:

  Ephedrine: mobile phase: methanol 0.08% and triethylamine solution = 18–82 (v/v), flow rate 1.0 mL/min, column temperature 25°C, UV detection wavelength 210 nm, and injection volume 10 *μ*L.
  Succinic acid: mobile phase: methanol 0.02 mol/L and KH2PO4 buffer solution = 7–93 (v/v), flow rate 1.0 mL/min, column temperature 25°C, ultraviolet detection wavelength 214 nm, and injection volume 10 *μ*L.
  6-gingerol and 6-shogaol: the mobile phases consisted of acetonitrile (A) and 0.1% formic acid (B) in a gradient elution: 0–10 min, 45% A; 10–15 min, 45–48% A; 15–17 min, 48–60% A; 17–43 min, 60% A; 43–45 min, 60–67% A; 45–48 min, 67–69% A; 48–58 min, 69–71% A. The flow rate was 0.5 mL/min, column temperature set at 30°C, ultraviolet detection wavelength 230 nm, and injection volume 20 *μ*L.

### 2.4. Preparation of Kaolin Pellets

Kaolin pellets were prepared according to the methods described by Takeda et al. with slight modifications [32]. Briefly, kaolin (china clay, hydrated aluminium silicate) was mixed with 2% gum arabic in distilled water to form a thick paste, which was then extruded through a syringe to produce rods with a similar diameter to that of the normal food pellets and dried at room temperature. The rods were then cut into pellets with a similar size to the normal food pellets.

### 2.5. Animals and Treatment

Male Wistar rats with a body weight of 200–220 g were purchased from Jinan Pengyue Experimental Animal Breeding Co., Ltd. (SCXK [LU] 2014–0007, Jinan, China). Rats were housed in a specific pathogen-free (SPF) environment maintained at 22°C ± 2°C and 55% ± 5% relative humidity under a 12/12 h circadian rhythm.

Twenty-four rats were randomized into four groups (six rats per group) as follows: normal control group, cisplatin model group, ondansetron-treatment group, and XBXT-treatment group. Rats were placed in individual cages and allowed access to both water and diet *ad libitum* during the 7-day adaptation period before drug administration. Kaolin was introduced into a separate compartment in the food hopper 3 days prior to the start of the experiment to allow the animals to be acclimatized to the presence of kaolin in the cage. At 8 : 00 on the day of the experiment, the normal control group received an intraperitoneal (i.p.) injection of saline (1 mL/100 g), while rats from the other three groups received a single dose of cisplatin (6 mg/kg). Ondansetron (1.3 mg/kg), XBXT (1.6 g/kg), or distilled water (1 mL/100 g) was administered by gastric gavage (i.g.) 1 h before the administration of cisplatin (or saline) and thereafter was administered every 12 h for 3 days. Kaolin and food intake as well as body weight were measured every 24 h until the end of the experiment. All animals were sacrificed at 72 h after cisplatin injection. Blood, ileum, and gastric antrum samples were obtained for further analysis. The experiments were performed in accordance with the policies and guidelines of the Ethic Committee for Animal Use in Guangdong Pharmaceutical University. The dose of XBXT was equal to 6.4-fold of the clinical dose (equivalent to adult dosage) based on our previous study showing that XBXT (3.2 g/kg·d^−1^) elicited remarkable anti-inflammatory and antiemetic effects in rats [33, 34]. The dose of cisplatin (6 mg/kg) was chosen based on previous studies showing reliable induction of pica and suppression of food intake [33–35].

### 2.6. Measurement of Food and Kaolin Intake and Body Weight

Kaolin and food consumption were monitored, and rats were weighed from 6 : 30 to 7 : 00 daily starting from the day of kaolin introduction. Any spillage of food and kaolin were collected and weighed, and food hoppers were then refilled and weighed.

### 2.7. Histological Analysis

The excised tissue from the ileum and gastric antrum was rapidly cleaned and fixed in 4% paraformaldehyde at 4°C for 24 h. After embedding in paraffin, blocks were cut into 4 *μ*m-thick sections using a rotary microtome (RM2235, Leica, Shanghai, China) and stained with hematoxylin-eosin (HE). Finally, each section was examined under a light microscope (Eclipse C1, Nikon, Tokyo, Japan) for evaluation of the histopathologic changes in the ileum and gastric antrum. Histopathological examination was done in a blinded fashion, analyzing five microscopic fields of each organ per animal.

### 2.8. ELISA Measurement

The levels of ROS and cytokines, including IL-1*β* and IL-18, in serum were determined using commercially available ELISA ROS, IL-1*β*, and IL-18 kits (Shanghai Enzyme-linked Biotechnology Co., Ltd., Shanghai, China) according to the manufacturer's protocols.

### 2.9. Western Blot Analysis

Ileum and gastric antrum samples were homogenized in 1 mL of RIPA lysis buffer (Beyotime Institute of Biotechnology, Shanghai, China) containing phenylmethanesulfonyl fluoride (Beyotime) and centrifuged at 12,000 rpm at 4°C for 20 min. The supernatants were collected, and the protein concentration was determined using a bicinchoninic acid (BCA) protein assay kit (Beyotime). Lysates containing equal amounts of protein were heated at 98°C in sodium dodecyl sulfate (SDS) sample buffer for 10 min and separated by 8 or 12% sodium dodecyl sulfate polyacrylamide gel electrophoresis (SDS-PAGE). Proteins were then electrotransferred onto a polyvinylidene difluoride (PVDF) membrane (Millipore, MA, USA). Membranes were blocked with Tris buffer saline-tween 20 (TBST) containing 5% skim milk for 2 h at room temperature and incubated with primary antibodies against caspase-1 (dilution 1 : 500; Santa Cruz Biotechnology, CA, USA), NLRP3 (dilution 1 : 1000; Abcam, Cambridge, UK), ASC (dilution 1 : 500; Abcam), IL-1*β* (dilution 1 : 5000; Abcam), GADPH (dilution 1 : 1000; Beyotime), and *β*-tubulin (dilution 1 : 5000; Beijing Emarbio Science and Technology Co., Ltd, Beijing, China) overnight at 4°C. Horseradish peroxidase- (HRP-) conjugated anti-mouse or anti-rabbit immunoglobulin G (IgG) was applied to membranes and incubated for 1 h at room temperature. Specific bands were detected with a chemiluminescence detection kit (ECL star; Beyotime) using a chemiluminescence imaging system (Sage Creation, Beijing, China). The intensity of the protein bands was quantitated using ImageJ software (NIH, Bethesda, MD, USA). GADPH and *β*-tubulin were used as the loading controls.

### 2.10. Immunofluorescence Analysis

Paraffin-embedded sections of the ileum and gastric antrum were dewaxed, rehydrated, and subjected to antigen heat retrieval in citrate buffer followed by blocking with 5% bovine serum albumin (BSA) for 30 min at room temperature. Next, sections were incubated with rabbit anti-NLRP3 (dilution 1 : 1500; Abcam, Cambridge, UK), anti-ASC (1 : 1000; Abcam, Cambridge, UK), and anti-caspase-1 (dilution 1 : 500; Santa Cruz Biotechnology, CA, USA) primary antibodies overnight at 4°C. After washing with PBS 3 times, slides were stained with Cy3 conjugated goat anti-rabbit IgG, FITC conjugated goat anti-rabbit IgG, or FITC conjugated goat anti-mouse IgG (Wuhan Servicebio Technology Co., Ltd., Wuhan, China) secondary antibodies for 1 h at room temperature. Sections were washed three times, and nuclei were stained with 4′,6-diamidino-2- phenylindole (DAPI) for 10 min at room temperature. Images were captured using a fluorescence microscope (Eclipse C1, Nikon, Tokyo, Japan). For colocalization analysis, all sections were taken randomly and analyzed using ImageJ software, and the summarized colocalization efficiency data were represented by Pearson's correlation coefficient (PCC) [36]. The fluorescence intensity of caspase-1 in each group was also measured by ImageJ software.

### 2.11. Statistical Analysis

Data were expressed as mean ± SEM. One-way analysis of variance (ANOVA) or two-way ANOVA was used to compare differences between multiple groups, and the results were analyzed by Tukey's multiple comparison test. *P* values <0.05 were considered statistically significant. Analyses were performed using Graph Pad Prism version 6.0 (GraphPad Software, San Diego, CA, USA).

## 3. Results

### 3.1. HPLC Fingerprinting of XBXT

**Figure 1:**
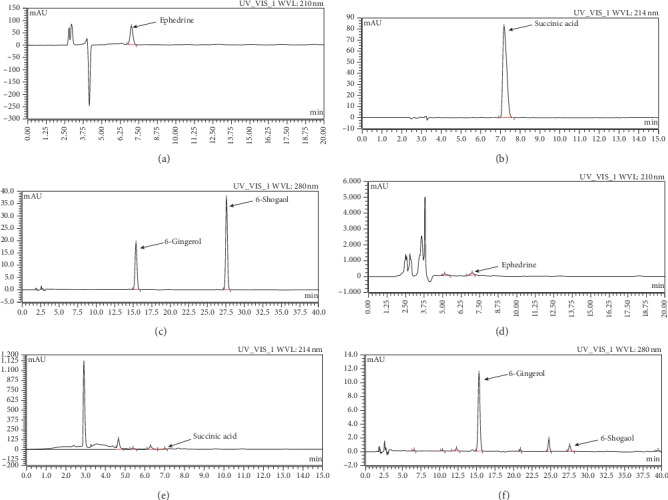
HPLC analysis of XBXT: (a–c) standard solutions and (d–f) extracts in XBXT.

The profile of the main components in XBXT was analyzed by HPLC. A representative chromatogram is shown in Figure 1. Ephedrine, succinic acid 6-gingerol, and 6-shogaol were well identified in XBXT based on their retention times and quantified using authentic standards (Table 2).

### 3.2. Effect of XBXT on Cisplatin-Induced Changes in Kaolin Consumption, Body Weight, and Food Intake

**Figure 2:**
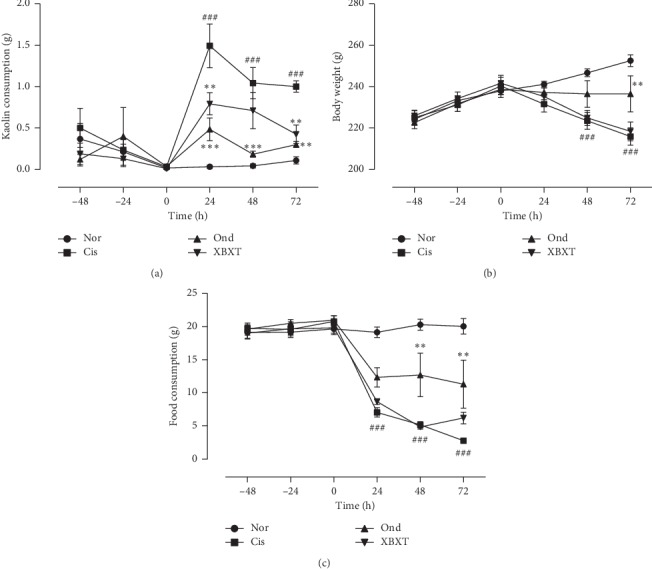
Effects of XBXT and ondansetron on kaolin ingestion (a), body weight (b), and food intake (c) in cisplatin-treated rats (*n* = 6). Cisplatin or vehicle (saline 0.9%w/v, 10 mL/kg, i.p.) was administered at *t* = 0. XBXT or ondansetron or distilled water was administered 1 h before cisplatin injection and then at regular 24 h intervals. Data represent the mean ± SEM. The data were analyzed for any significant differences using two-way ANOVA followed by Tukey's post hoc test. ^#^*P* < 0.05 and ^###^*P* < 0.001 vs the normal control group; ^*∗*^*P* < 0.05, ^*∗∗*^*P* < 0.01, and ^*∗∗∗*^*P* < 0.001 vs the cisplatin model group. Nor, normal control group; Cis, cisplatin model group; Ond, ondansetron treatment group; XBXT, XBXT treatment group.

Rats ate a small amount of kaolin on the first day of the accommodation period, but few animals ate any kaolin during the following days. Cisplatin treatment increased kaolin consumption during the 0–24 h (*P* < 0.001) period and the 24–72 h period (*P* < 0.001), which could be inhibited by ondansetron (*P* < 0.01). XBXT had a significant effect on kaolin consumption during the 0–24 h (*P* < 0.01) period and the 48–72 h period (*P* < 0.01) in cisplatin-treated animals. Cisplatin significantly decreased body weight in a time-dependent manner compared to normal control animals during the 24–72 h period (*P* < 0.001). There was a trend towards mitigation of the reduction in body weight during the 48–72 h (*P* < 0.001) period after ondansetron administration compared with cisplatin-treated rats. The administration of cisplatin significantly reduced food intake compared to that in normal control group rats throughout the observation period (*P* < 0.001). Compared to cisplatin-treated rats, ondansetron administration tended to increase food intake during the 24–72 h (*P* < 0.01) period. However, the reduction in food intake was not significantly ameliorated in XBXT-treated rats, both during the 0–24 h period and the 24–72 h period (Figure 2).

### 3.3. Effect of XBXT on Histological Damage

**Figure 3:**
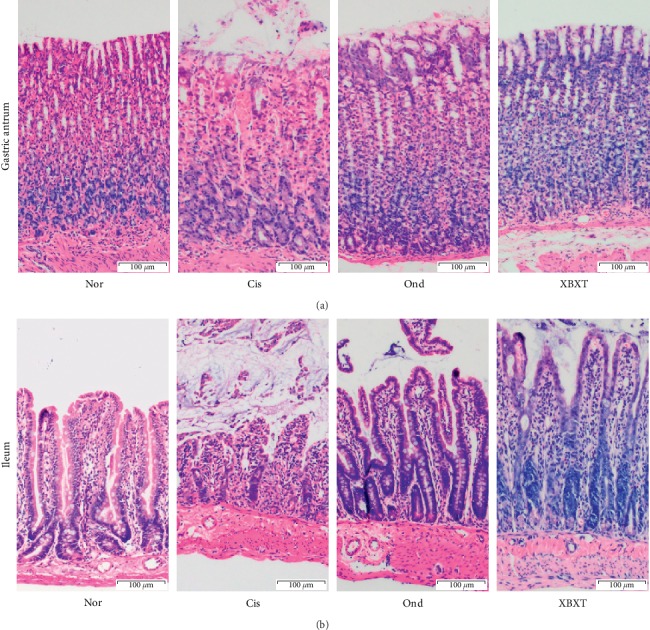
Effect of XBXT and ondansetron on histopathological changes in the rat gastric antrum (a) and ileum (b) induced by cisplatin (*n* = 3). At 72 h after administration of cisplatin or normal saline, the dissected antrum and ileum were fixed with paraformaldehyde (4%) and then embedded in paraffin. Sections were stained with HE. All sections, original magnification ×100. Scale bar = 100 *μ*m. Nor, normal control group; Cis, cisplatin model group; Ond, ondansetron treatment group; XBXT, XBXT treatment group.

As shown in Figure 3, severely injured epithelial mucosa was seen in the ileum at 72 h after cisplatin administration, whereas evidence of mucosal injury was minimal in the normal control group. Additionally, cisplatin treatment resulted in a massive inflammatory reaction characterized by increased inflammatory cell infiltration in both mucosal and submucosal regions. Massive deformities of villi including shortening of stature, loss, and atrophy, were also observed in cisplatin-treated animals. Administration of XBXT or ondansetron attenuated these histological deformities.

In the gastric antrum, the epithelial cells on the surface of the gastric mucosa were destroyed, the lamina propria showed inflammatory cell infiltration, and the outer layer of the mucosa peeled off after cisplatin administration. XBXT or ondansetron attenuated these histological deformities. There was no obvious pathological change in the normal control group.

### 3.4. Effect of XBXT on ROS and Proinflammatory Cytokine Level in Rat Serum

**Figure 4:**
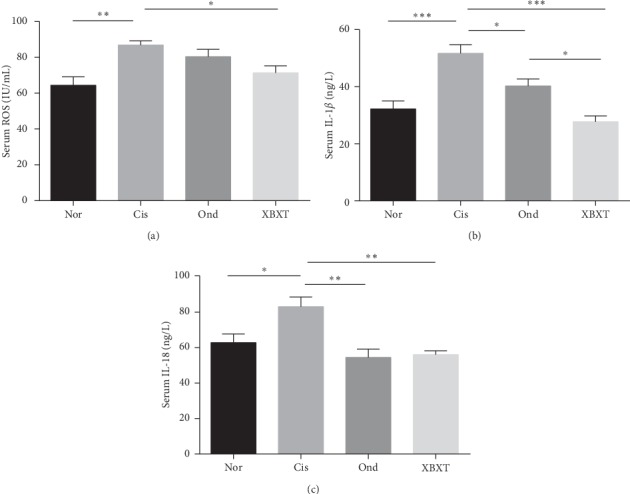
Effect of XBXT and ondansetron on the expression of ROS, IL-1*β*, and IL-18 induced by cisplatin in the rat serum (*n* = 6). Serum of rats was tested by ELISA for (a) ROS, (b) IL-1*β*, and (c) IL-18. Data represent the mean ± SEM. The data were analyzed for significant differences using one-way ANOVA followed by Tukey's post hoc test. ^*∗*^*P* < 0.05, ^*∗∗*^*P* < 0.01, and ^*∗∗∗*^*P* < 0.001. Nor, normal control group; Cis, cisplatin model group; Ond, ondansetron treatment group; XBXT, XBXT treatment group.

ROS and inflammatory cytokines in serum were assessed by ELISA (Figure 4). The results showed that cisplatin administration significantly induced an increase in ROS, IL-1*β*, and IL-18 levels in serum compared to the normal control group (*P* < 0.05). As expected, both ondansetron and XBXT treatment significantly downregulated the expression of IL-1*β* and IL-18 compared with the cisplatin model group (*P* < 0.05). XBXT, but not ondansetron, reduced ROS levels (*P* < 0.05).

### 3.5. Effect of XBXT on NLRP3 Inflammasome-Related Protein Expression in the Rat Gastric Antrum and Ileum

**Figure 5:**
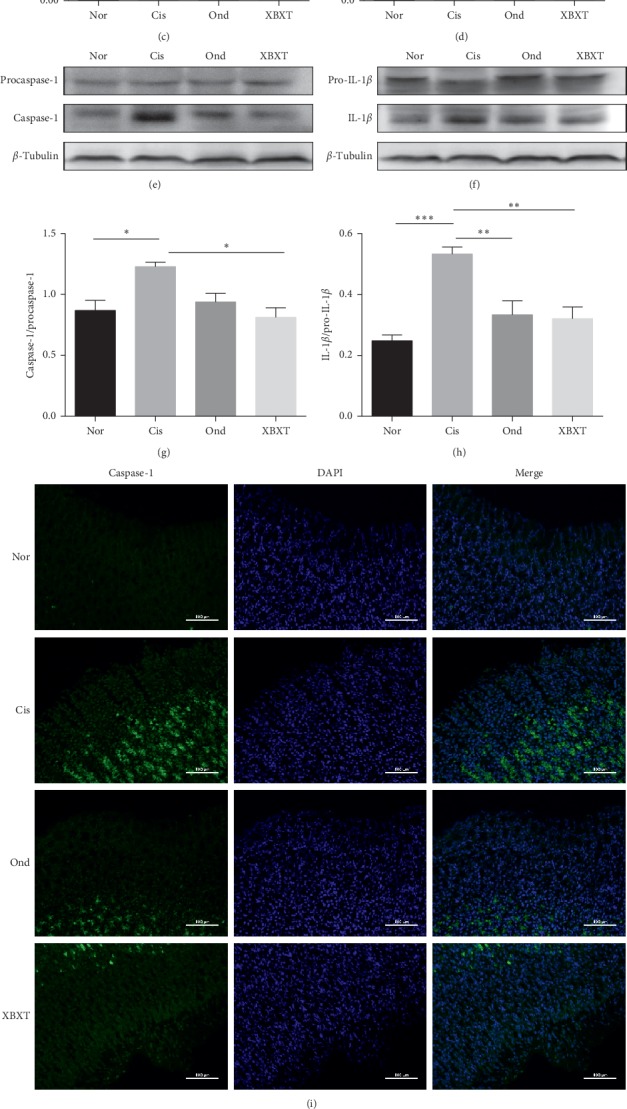
Effect of XBXT and ondansetron on NLRP3 inflammasome protein expression induced by cisplatin in the rat antrum (*n* = 3). (a–h) Western blot analysis of NLRP3, ASC, caspase-1, and IL-1*β* in the antrum, and band densities were converted to a bar graph. (i) Representative photograph of caspase-1 with immunofluorescence staining of the antrum, and (j) quantitative data showing the mean fluorescence intensity of caspase-1. Photographs were observed under a fluorescence microscope at 200 ×magnification (scale bar = 100 *μ*m). Data represent means ± SEM. The data were analyzed for significant differences using a one-way ANOVA followed by Tukey's post hoc test. ^*∗*^*P* < 0.05, ^*∗∗*^*P* < 0.01, and ^*∗∗∗*^*P* < 0.001 vs the cisplatin model group. Nor, normal control group; Cis, cisplatin model group; Ond, ondansetron treatment group; XBXT, XBXT treatment group.

**Figure 6:**
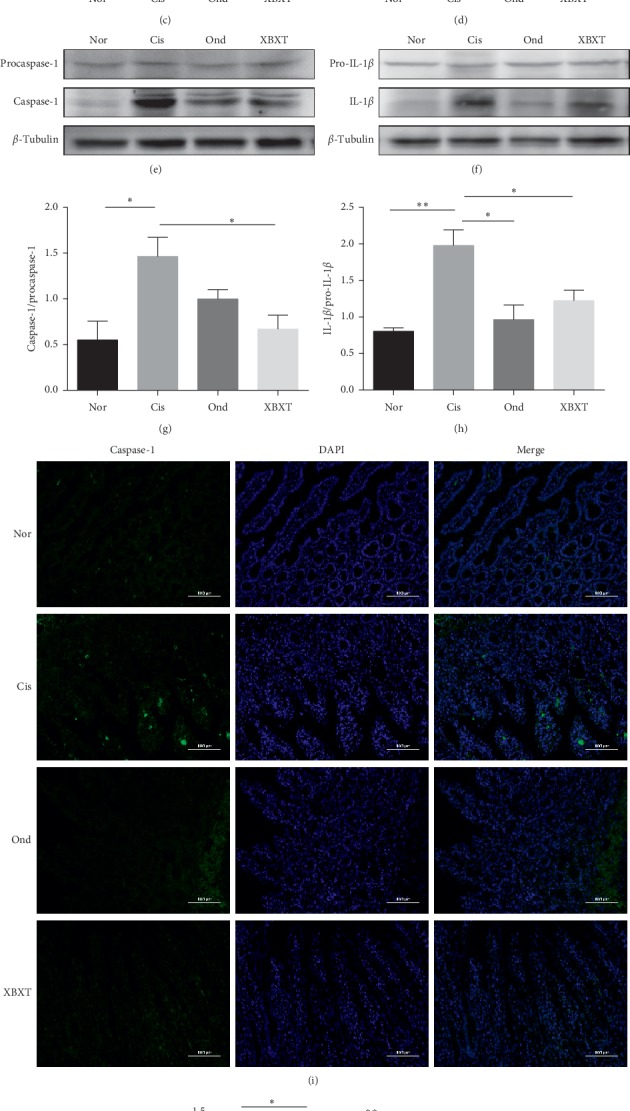
Effect of XBXT and ondansetron on NLRP3 inflammasome protein expression induced by cisplatin in the rat ileum (*n* = 3). (a–h) Western blot analysis of NLRP3, ASC, caspase-1, and IL-1*β* in the ileum. Band densities were converted to a bar graph. (i) Representative photograph of caspase-1 with immunofluorescence staining of the ileum, and (j) quantitative data showing the mean fluorescence intensity of caspase-1. Photographs were observed under a fluorescence microscope at 200 ×magnification (scale bar = 100 *μ*m). Data represent means ± SEM. The data were analyzed for significant differences using a one-way ANOVA followed by Tukey's post hoc test. ^*∗*^*P* < 0.05, ^*∗∗*^*P* < 0.01, and ^*∗∗∗*^*P* < 0.001 vs the cisplatin model group. Nor, normal control group; Cis, cisplatin model group; Ond, ondansetron treatment group; XBXT, XBXT treatment group.

To explore the possible molecular mechanism of ondansetron and XBXT, the expression of NLRP3 inflammasome-related proteins in the rat ileum and gastric antrum tissue was measured. As shown in Figures 5 and 6, cisplatin produced a significant increase in NLRP3, ASC, caspase-1, and IL-1*β* protein expression in the ileum and gastric antrum (*P* < 0.05). Ondansetron and XBXT inhibited these changes to different degrees. XBXT significantly inhibited cisplatin-induced increases in the protein level of NLRP3, ASC, caspase-1, and IL-1*β* both in the ileum and gastric antrum (*P* < 0.05). Ondansetron only significantly downregulated cisplatin-induced IL-1*β* levels in the ileum and gastric antrum (*P* < 0.05) and NLRP3 levels in the gastric antrum (*P* < 0.01). Caspase-1 is the functional executor component within the NLRP3 inflammasome [37]. We investigated the localization and expression level of caspase-1 in the ileum and gastric antrum using immunofluorescence. Caspase-1 immunofluorescence increased in the cisplatin-treated rats compared with the control group (*P* < 0.05) but was reduced upon XBXT treatment (*P* < 0.05). The reduction in the ondansetron treatment group was not statistically significant (Figures 5(i), 5(j), 6(i), and 6(j)). Caspase-1 was mainly localized to the cytoplasm upon exposure to cisplatin, and caspase-1 expression was observed in the lamina propria of the ileum and stomach mucosa.

### 3.6. Effect of XBXT on NLRP3 Inflammasome Formation in the Rat Gastric Antrum and Ileum

**Figure 7:**
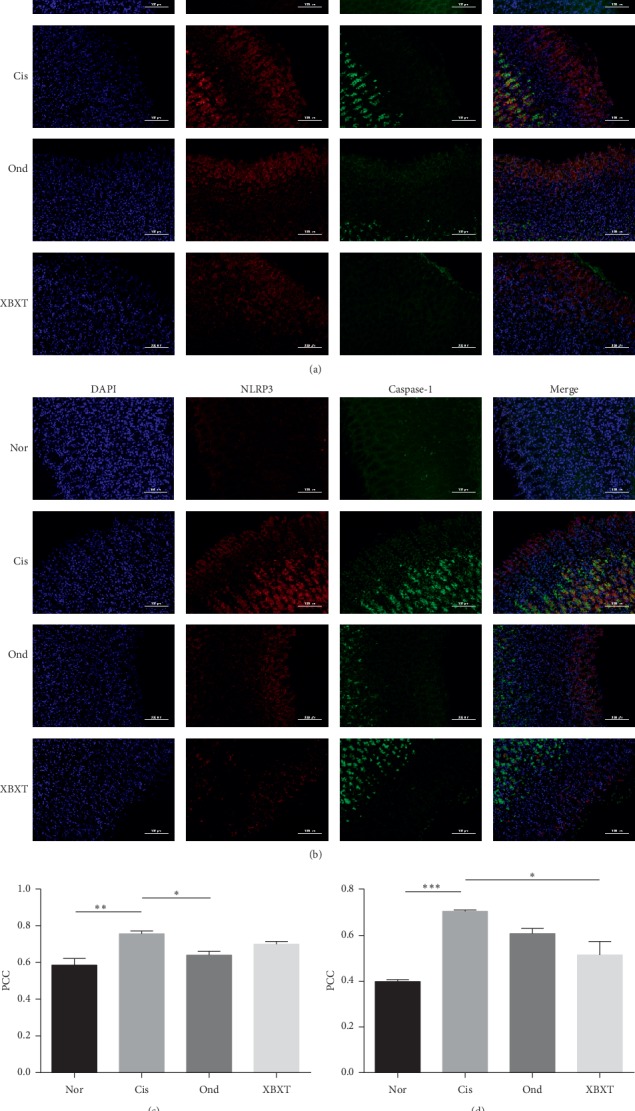
Effect of XBXT and ondansetron on NLRP3 inflammasome formation induced by cisplatin in the rat antrum (*n* = 3). (a) The colocalization images of NLRP3 (red)/ASC (green), (b) the colocalization images of NLRP3 (red)/caspase-1 (green), (c) summarized data showing the colocalization efficiency (Pearson's correlation coefficient) of NLRP3 with ASC, and (d) summarized data showing the colocalization efficiency (Pearson's correlation coefficient) of NLRP3 with caspase-1. Nuclei were stained with DAPI (blue). Photographs were observed under a fluorescence microscope at 200 × magnification (scale bar = 100 *μ*m). Data represent means ± SEM. The data were analyzed for significant differences using a one-way ANOVA followed by Tukey's post hoc test. ^*∗*^*P* < 0.05, ^*∗∗*^*P* < 0.01, and ^*∗∗∗*^*P* < 0.001 vs the cisplatin model group. Nor, normal control group; Cis, cisplatin model group; Ond, ondansetron treatment group; XBXT, XBXT treatment group.

**Figure 8:**
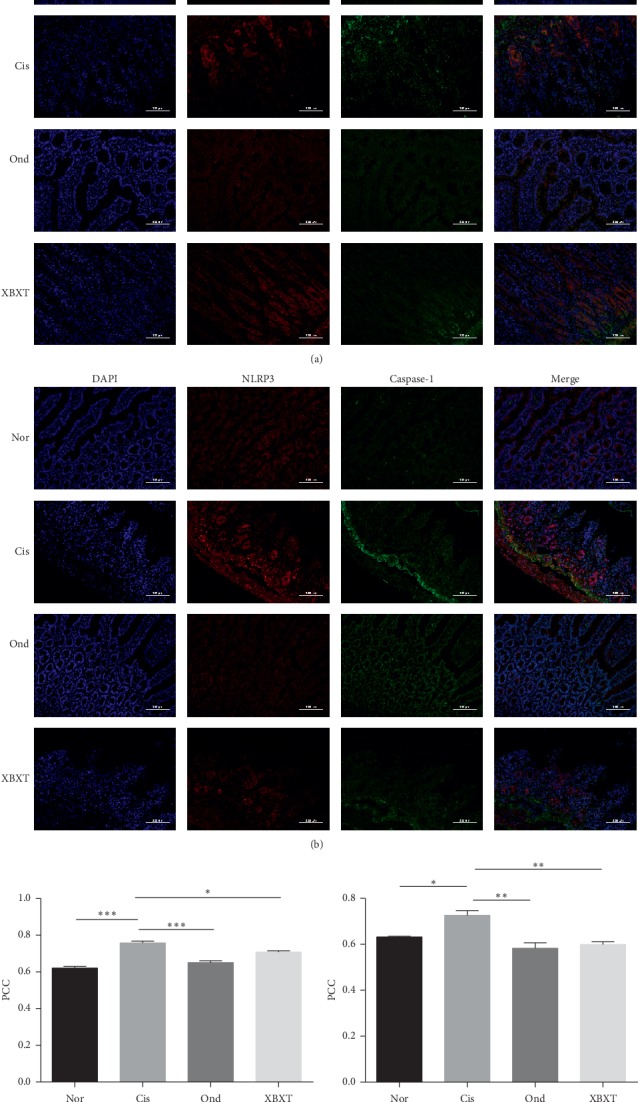
Effect of XBXT and ondansetron on NLRP3 inflammasome formation induced by cisplatin in the rat ileum (*n* = 3). (a) The colocalization images of NLRP3 (red)/ASC (green), (b) the colocalization images of NLRP3 (red)/caspase-1 (green), (c) summarized data showing the colocalization efficiency (Pearson's correlation coefficient) of NLRP3 with ASC, and (d) summarized data showing the colocalization efficiency (Pearson's correlation coefficient) of NLRP3 with caspase-1. Nuclei were stained with DAPI (blue). Photographs were observed under a fluorescence microscope at 200 × magnification (scale bar = 100 *μ*m). Data represent means ± SEM. The data were analyzed for significant differences using a one-way ANOVA followed by Tukey's post hoc test. ^*∗*^*P* < 0.05, ^*∗∗*^*P* < 0.01, and ^*∗∗∗*^*P* < 0.001 vs the cisplatin model group. Nor, normal control group; Cis, cisplatin model group; Ond, ondansetron treatment group; XBXT, XBXT treatment group.

The colocalization of NLRP3 with ASC or caspase-1 is shown in Figures 7 and 8. The colocalization of NLRP3 and ASC or caspase-1 significantly increased in cisplatin-treated rats (*P* < 0.05), but not in the normal control group. This suggests the formation of NLRP3 inflammasomes in the ileum and gastric antrum in response to cisplatin. Surprisingly, we found that both ondansetron and XBXT decreased this inflammasome formation in the ileum and gastric antrum. However, in antrum tissue, the inhibitory effect of XBXT on the cisplatin-induced increase in the colocalization of NLRP3 and ASC was not significant. Ondansetron did not block the colocalization of NLRP3 and caspase-1 induced by cisplatin in the antrum.

## 4. Discussion

CINV can be a serious problem for cancer patients receiving oncological treatment due to the inherent emetogenicity of chemotherapeutic agents [38]. The mechanism of emesis is a complex multifactorial process, but investigations have shown that emesis may be primarily mediated by several neurotransmitters in the gastrointestinal and central nervous systems [39]. The main mechanism of acute emesis is thought to be the stimulation of the vomiting center of the medulla oblongata via stimulation of 5-HT_3_Rs on the vagal afferent terminals in the wall of the gut by 5-HT, which is released from enterochromaffin cells (EC cells) lining the upper intestine after cisplatin administration. The mechanism of delayed emesis has been regarded as largely associated with the activation of neurokinin 1 receptors by substance P [2]. Alternatively, it has been hypothesized that emesis is the result of bowel inflammation [10].

In the mid-1980s, Miner and Sanger demonstrated that a selective 5-HT_3_RA could attenuate cisplatin-induced emesis in ferrets [40]. Acute emesis is well prevented by the use of selective 5-HT_3_RAs such as ondansetron [41]. However, 5-HT_3_RAs also plays a major role in delayed emesis [42]. Several reports have shown that improved control of delayed emesis is achieved by a combination of 5-HT_3_RA and dexamethasone in both human patients and animals [43, 44], or by dexamethasone alone [10]. Furthermore, anti-inflammatory drugs, including meloxicam and dexamethasone, can ameliorate opposing enzymatic changes in ileal 5-HT metabolism after cisplatin administration in rats [13]. These studies indicate that there is an interaction between the 5-HT/5-HT_3_R system and the inflammatory response in emesis.

Most studies show that cytotoxic therapy leads to the generation of ROS [19, 45]. ROS are known to cause damage to cells and tissues and activate the NLRP3 inflammasome and the NF-*κ*B signaling pathway, which promotes the upregulation of key cytokines such as IL-1*β* [46, 47]. Moreover, studies have shown that IL-1*β* can stimulate 5-HT secretion from EC cells in the gastrointestinal mucosa [48]. Thus, IL-1*β* signaling may underlie CINV, and the inflammatory response is a potent factor resulting in the pathogenesis of CINV. Accordingly, we investigated the relationship between the gastrointestinal tract inflammatory response to cisplatin and CINV in a rat pica model.

Although cisplatin is the most commonly used antineoplastic drugs in the treatment of many patients with solid organ cancer, it has strong side effects on the gastrointestinal tract [49]. As reported previously, cisplatin at a dose of 6 mg/kg induced pica within 8 hours after administration of the drug, and these behaviors continued for 5 days [35]. In addition, Rudd et al. discovered that cisplatin induced acute and delayed kaolin ingestion at a dose of 3 mg/kg [50]. We confirmed that pica behavior in both the acute and delayed phase increased after cisplatin administration at a dose of 6 mg/kg. Importantly, we also observed that cisplatin markedly reduced body weight and food intake. Similar observations have been made with the use of cisplatin in other experimental studies [50, 51]. Cisplatin-induced gastrointestinal toxicity involves various complex multifactorial processes, among which inflammation and oxidative stress are regarded as important factors [8, 52]. The production of oxidative stress is accompanied by an increase in ROS. We examined cisplatin-induced ROS levels in rats treated with XBXT or ondansetron. The results showed that ROS levels in the cisplatin model group were significantly increased compared with those in the normal control group, and that XBXT significantly decreased the cisplatin-induced increase in ROS.

According to previous studies, ROS serves as the trigger of the NLRP3 inflammasome [53]. Cisplatin activates NLRP3 inflammasome expression and releases proinflammatory cytokines, thereby resulting in acute injury to the liver and kidneys in rats [22]. Several cytokines, such as IL-1*β*, are elevated in the small intestinal tissue and serum in response to cisplatin, resulting in distinct histopathological changes in the gastrointestinal tract [54]. Thus, we suggest that there is an NLRP3 inflammasome-dependent inflammatory response mediating cisplatin-induced side effects in the gastrointestinal tract. In the present study, we observed that the expression of NLRP3, ASC, and caspase-1, which constitute the NLRP3 inflammasome, was increased in cisplatin-treated rats. The proinflammatory cytokines IL-1*β* and IL-18 were upregulated after cisplatin treatment alongside severe damage to the ileum and gastric antrum. Importantly, the colocalization of NLRP3 and ASC and that of NLRP3 and caspase-1 was markedly enhanced in the ileum and gastric antrum. Taken together, these results indicate that the NLRP3 inflammasome signaling pathway may be an important determinant of cisplatin-induced CINV.

Ondansetron, a first-generation 5-HT_3_RA, has been widely effective against acute emesis [55]. However, ondansetron alone has only moderate efficacy in the control of cisplatin-induced delayed emesis in humans and experimental animals [56–58]. In the current study, we found that ondansetron reduced cisplatin-induced kaolin consumption both in the acute and delayed phases. Recently, increasing evidence has suggested that 5-HT_3_RAs possess anti-inflammatory properties. Indeed, 5-HT_3_RAs, including ramosetron and ondansetron, have been shown to ameliorate 5-FU-induced intestinal mucositis in mice, and that this action could result from suppression of apoptotic responses in the intestinal crypt cells via inhibition of cytokine expression [12]. Motavallian-Naeini et al. reported that ondansetron healed colonic macroscopic and histological damage and decreased levels of proinflammatory cytokines, including IL-1*β* in the trinitrobenzene sulfonic acid (TNBS) model of rat colitis [59]. The experiments in this paper confirmed that ondansetron treatment attenuates increases in IL-1*β* and IL-18 levels induced by cisplatin. This further indicates that the antiemetic effect of ondansetron may be related to the inhibition of inflammatory activity. It is worth noting that 5-HTRs have been found in inflammatory cells such as macrophages, a principle source of proinflammatory cytokines IL-1*β* [60, 61]. In view of the above, the inhibition effects of ondansetron upon proinflammatory cytokines in cisplatin-induced rats are at least partly mediated through its effects in a receptor-dependent fashion. Release of cytokines by intestinal immunocytes can activate neighbouring EC cells to secrete 5-HT [48]. This increased 5-HT secretion results in activation of more 5-HT_3_Rs in the gut (EC cells or vagal nerve terminals) [62]. Thus, ondansetron may be useful for preventing not only nausea and emesis, but also the intestinal inflammatory response during chemotherapy. In summary, ondansetron attenuated CINV partially through its anti-inflammatory action, but this action may be independent of the NLRP3 inflammasome pathway.

XBXT is a traditional Chinese herbal formulation and has been used to treat emesis, but the precise mechanism of XBXT in emesis remains unknown. The present study revealed that XBXT inhibited NLRP3 inflammasome activation and subsequent IL-1*β* and IL-18 release in response to cisplatin treatment. XBXT weakened the pica behavior stimulated by cisplatin. The above results indicate that XBXT alleviates cisplatin-induced CINV, which may be related to the suppression of NLRP3 inflammasome activation.

## 5. Conclusion

In conclusion, the above results reveal that XBXT possesses a protective effect against CINV, possibly mediated via amelioration of oxidative stress, reduction of the release of inflammatory factors including IL-1*β* and IL-18, and inactivation of the NLRP3 inflammasome. Overall, our results suggest that XBXT may be a promising additional therapy to treat CINV.

## Figures and Tables

**Table 1:** The composition of XBXT.

TCM materials (pinyin)	English name	Latin name	Part used	Voucher specimen number	Dry weight (g) of daily dose in clinic
Ban xia	Pinellia	*Pinellia ternata* (Thunb.) Breit.	Rhizome	J7654	20
Sheng jiang	Ginger	*Zingiber officinale* Rosc.	Rhizome	J7201	10

**Table 2:** Herbal sources and retention times of components in XBXT.

Constituents	Source	Retention times (min)	Concentration in XBXT (mg/g)
Ephedrine	*Pinellia ternata* (Thunb.) Breit.	6.993	0.309
Succinic acid	*Pinellia ternata* (Thunb.) Breit.	7.012	0.025
6-Gingerol	*Zingiber officinale* Rosc.	15.337	0.0616
6-Shogaol	*Zingiber officinale* Rosc.	27.563	0.0025

## Data Availability

The data used to support the findings of this study are available from the corresponding author upon request.
